# Medically Important Parasites Carried by Cockroaches in Melong Subdivision, Littoral, Cameroon

**DOI:** 10.1155/2017/7967325

**Published:** 2017-08-20

**Authors:** R. J. Atiokeng Tatang, H. G. Tsila, J. Wabo Poné

**Affiliations:** Research Unit of Biology and Applied Ecology, Department of Animal Biology, Faculty of Science, University of Dschang, P.O. Box 067, Dschang, Cameroon

## Abstract

Cockroaches have been recognized as mechanical vectors of pathogens that can infest humans or animals. A total of 844 adult cockroaches (436 males and 408 females) were caught. In the laboratory, cockroaches were first washed in saturated salt solution to remove ectoparasites and then rinsed with 70% alcohol, dried, and dissected for endoparasites. An overall transport rate of 47.39% was recorded. Six genera of parasites were identified. These were* Ascaris* (33.76%),* Trichuris* (11.97%),* Capillaria* (6.16%),* Toxocara* (4.86%), Hook Worm (4.86%), and* Eimeria *(2.73%). The parasites were more recorded on the external surface (54.27%) of cockroaches than in the internal surface (GIT, 38.51%). The same tendency was obtained between sexes with female cockroaches having a higher transport rate (36.69%). Cockroaches caught in toilets carried more parasites (31.99%) as compared to those from kitchens (22.63%) and houses (11.14%). Almost all encountered parasites were recognized as responsible of zoonosis and they can be consequently released in nature by hosts and easily disseminated by cockroaches as mechanical vectors. Sanitary education, reenforcement of worms' eradication programs, and the fight against these insects remain a necessity in the Mélong Subdivision.

## 1. Introduction

Cockroaches have survived on the earth for more than 300 million years virtually without involving change [1]. They are considered one of the most successful groups of animals because of their adaptability in various environmental conditions. There are approximately 3500 species of cockroaches worldwide [2]. Among these species, thirty are associated with human habitations [3].* Periplaneta americana, *Linnaeus, 1758, and* Blattella germanica, *Linnaeus, 1767, are the most common species [4, 5]. The majority of these species lives in tropical and subtropical areas where they are not recognized as pests [6]. In these areas, they are abundantly found in areas with frequent stagnant water bodies or with a constant and high moisture availability such as toilets, kitchens, sewages, and drainages where water serves as migration routes from place to place [7]. In addition to their repulsive and annoying characteristics, they eat and contaminate food and leave a persistent offensive odor in infested places [1]. Cockroaches frequently feed on human faeces, garbage, and sewage [8]. Therefore they have copious opportunities to disseminate pathogenic agents on food resources. They are nocturnal and have filthy habits which coupled with their feeding mechanisms make them efficient vectors of pathogens like bacteria (*Klebsiella pneumonia, Enterobacter cloacae, Enterobacter aerogenes, Salmonella *spp.*, Shigella sonnei, Vibrio cholerae, Citrobacter freundii *[9], viruses* (Poliomyelitis)*) [10], protozoa (oocysts of* Isospora belli, Cryptosporidium parvum, Cyclospora cayetanensis, *cysts of* Entamoeba histolytica, Balantidium coli, *and* Giardia lamblia*) [8, 11–13], fungi (*Candida *sp.*, Rhizopus *sp.*, Aspergillus *sp.*, Mucor *sp.) [14], and eggs of some pathogenic intestinal worms (*Ascaris lumbricoides, Trichuris trichiura, *Hookworm,* Enterobius vermicularis, Hymenolepis nana, Toxocara canis, and Strongyloides stercoralis *larvae) [15–17]. Cockroaches not only contaminate food with their droppings or by pathogens but they also cause food poisoning [18]. According to Tatfeng et al. [14], some people are allergic to antigens and faeces of cockroaches which may result in asthmatic–related health problems. Studies have been carried out in several countries of the world in order to evaluate the risks of parasitic infestation associated with the presence of cockroaches in households. In Cameroon, to the best of our knowledge, no or few epidemiological data on this risk factor associated with the presence of cockroaches in the households are available. The present study was therefore designed to identify cockroach species while examining the occurrence of medically important parasites they carried in the Melong Subdivision, Littoral Region, Cameroon. The findings may be of immense benefit to the local residents and for others within and outside Cameroon as it will serve as an educational tool for the potential dangers they might face with the presence of cockroaches in their houses.

## 2. Materials and Methods

### 2.1. Study Area

The study was carried out in Melong Subdivision of the Moungo Division, Littoral Region of Cameroon, with the following coordinate at Latitude 5°07′22′′ North and Longitude 9°57′08′′ East. The locality experiences two rainy seasons: the longer one runs from June 20th to November 15th and a shorter one goes from March 20th to April 15th with an annual average rainfall of 1960 mm and two dry seasons from November 20th to March 15th for the larger one and April 20th to May 15th for the shorter one. Average temperature ranges from 22.5°C to 24°C. People of Melong Subdivision are predominately peasant farmers. Their sanitary conditions are below standard. Due to the lack of pipe borne water supply, they depend sufficiently on spring and well water for most of their occupational and domestic activities. With no toilet facilities, a majority use man-hole toilets while others defecate on road sides and bushes and refuse dumps. Their system of animal husbandry is underdeveloped. So, they are frequently in contact with animals like pigs, dogs, goats, and cats. These facilities largely contribute to the proliferation of cockroaches which are reported to be serious vectors of pathogens.

### 2.2. Sample Collection

A total of eight hundred and forty-four (844) adult cockroaches were caught in three-quarters (Nkongsoung, Melong Centre and Lèlem) of Melong Subdivision in the night from 63 households randomly selected between 8.00 pm and 11.00 pm in toilets, kitchens, and houses using sterile hand gloves. Each cockroach was preserved in labeled sterile vial containing cotton soaked in diluted 10% chloroform. Containers were transported in an ice back to the laboratory for identification and parasitological analyses.

### 2.3. Identification of Cockroaches and Parasitological Analysis

Once in the laboratory, cockroaches were identified according to their morphological and morphometric characteristics using standard taxonomic keys [19]. For ectoparasites analysis, 32 ml of saturated salt solution (40%) was added to the vial containing the cockroach. The vial was shaken for 2 min to remove attached parasites [16]. Then, 16 ml of the suspension was transferred in two test tubes until an upper meniscus was formed. Immediately, cover slides were carefully placed on top of the tubes. The preparation was allowed to stand for 10 min to allow parasitic stages to float and fix on the slides. Then, cover slides were delicately removed and placed on a slide and the preparation was examined using light microscope at 10x for the identification of parasites [20]. For cysts and oocysts identification, one drop of 1% Lugol's iodine was added at the border of the cover slides and the preparation was observed at 40x. As far as internal parasites were concerned, washed cockroaches were individually placed in flask and rinsed with 70% alcohol. They were then transferred into sterilized flake and allowed to dry at room temperature (25°C) and then washed with normal saline for 2-3 min. After washing, each cockroach was fixed in a Petri dish. The head and legs were isolated and the abdomen was opened using fine pointed forceps with small scissors. The gut and other internal organs were removed using fine needles. The GIT of the cockroach was removed, opened, and washed in a vial with 32 ml of saturated salt solution (40%). This was homogenized and filtered using a tea sieve. About 20 ml of the filtrate was used to fill a test tube until an upper meniscus was formed. Then the same procedure was followed as described earlier.

### 2.4. Statistical Analyses

Descriptive statistics were used to determine the transport rate while Chi-squared (*χ*^2^) analysis was used to determine association and significant differences between the parameters tested at *p* < 0.05.

## 3. Results

### 3.1. Distribution of Cockroaches per Quarters and Capture Sites

A total of 844 adult cockroaches were collected from 63 households randomly selected during the period of survey. Three species of cockroach,* Periplaneta americana, Blattella germanica, and Blatta orientalis *were identified (Table 1). Almost all the species were found in the three-quarters of the study area.

All the species of cockroach identified were found in all capture sites. Irrespective of the capture sites, the most prevalent species was* P. americana* (58.41%), followed by* B. germanica* (21.45%) and* B. orientalis* (20.14%) (Table 1). Out of the cockroaches encountered,* P. americana* had the highest prevalence in toilets (33.80%), as compared to kitchens (15.62%) and houses (9.48%).* Blattella germanica* was most prevalent in houses (14.10%) and kitchens (7.35%). This species was absent in toilets.* Blattella orientalis* was also most prevalent in houses (10.19%) followed by kitchens (9.95%) and absent in toilets.

### 3.2. Cockroaches Parasitic Transport Rate

Out of 844 cockroaches caught, 400 (47.39%) were found to be carriers of nematodes eggs and protozoan oocysts. From these cockroaches, 256 (30.33%) were* P. americana* followed by 76 (9.00%)* B. germanica* and 68 (8.06%)* B. orientalis.*

### 3.3. Species of Parasites Identified

**Figure 1:**
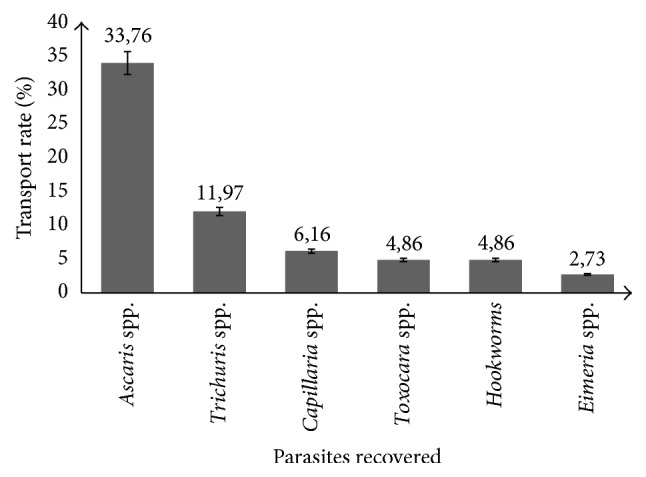
Transport rate of various parasites isolated from the cockroaches. Error bars = 95% CI.

The different medically important parasites isolated from external and internal surfaces of cockroaches are presented in Figure 1. It is found that the most prevalent medically important parasitic stage identified was* Ascaris *spp. eggs (33.76%) followed by that of* Trichuris *spp. (11.97%) and* Capillaria *spp. (6.16%). Eggs of* Toxocara *spp. and Hookworm have the same transport rate (4.86%).* Eimeria *spp. oocysts (2.73%) were less prevalent.

### 3.4. Predilection Sites of Infestation and Sex-Related Frequency

The prevalence of medically important parasites in relation to their repartition on cockroach is presented on Table 2. All the six parasites identified were more prevalent in the external surface (54.27%) as compared to the internal surface (GIT; 38.51%).* Ascaris *spp. eggs had the highest prevalence (49.64%), followed by* Trichuris *spp. (16.46%)* Capillaria *spp. (9.12%),* Toxocara *spp. (7.82%), Hookworms (4.98%) ones, and* Eimeria *spp. oocysts (4.74%).

The transport rate of medically important parasites identified in relation to cockroach sexes is illustrated in Table 2. Female cockroaches were more common carriers of parasites than the males.* Ascaris *spp. eggs were more carried (33.76%), followed by those of* Trichuris *spp. (11.97%),* Capillaria *spp. (6.16%),* Toxocara *spp. (4.86%), Hookworms (4.86%), and oocysts of* Eimeria *spp. (2.73%).

### 3.5. Parasite Recovery Rate from Cockroaches in Different Quarters and Capture Sites

The prevalence of identified parasite in relation to the quarter is shown in Table 3. Cockroaches in Lèlem carried more parasites (26.07%) followed by those caught in Nkongsoung (20.97%) and in Melong centre (17.29%). Irrespective of quarters,* Ascaris *spp. eggs were more carried (33.77%) followed by those of* Trichuris *spp. (11.97%),* Capillaria *spp. (6.16%),* Toxocara *spp. (4.86%), Hookworm (4.86%), and* Eimeria *spp. (2.73%) oocysts.

The transport rate of medically important parasites identified in relation to the capture sites is shown in Table 3. This study revealed that cockroaches trapped from different sites (toilets, kitchens, and houses) shared the same parasites except for* Toxocara spp.* and* Eimeria spp.* which were absent on cockroaches caught in toilets. Cockroaches trapped in the toilets carried more medically important parasites (32.37%) as compared to those from kitchens (22.90%) and those from houses (11.27%).

### 3.6. Prevalence of Parasitic Association

Four types of parasitic associations were recorded from the infested cockroaches. Single parasitic infestation was the most frequent (32.58%) followed by double (11.37%), triple (1.89%), and quadruple (0.24%) infestations. No cockroach had five parasites.

## 4. Discussion

Speculations have always been made on the involvement of cockroaches as possible vectors of diseases in communities [17]. But their role in the direct transmission of pathogens has seldom been established [16, 17]. Findings from this study indicated that cockroaches are reservoirs and mechanical transmitters of parasites. This is in agreement with previous studies done by Nagham et al. [15]; Bala and Sule [16] in Sokoto; Kassiri and Kazemi [21]; and Etim et al. [17], respectively, in Iraq, Iran, and Calabar, Nigeria.

Three species of cockroaches (*Periplaneta americana*,* Blattella germanica, *and* Blatta orientalis*) were found to be dominant in human habitations in the Melong Subdivision. This could be mainly due to their cosmopolitan distribution (association with human activity such as global commerce) and to their ability to reproduce and to survive more easily in tropical climate region. Although Chan et al. [22], Al-Mayali and Al-Yaqoobi [4], Ajero et al. [23], Ejimadu et al. [24], and Nigeria and Ameh [25], respectively, in Hawaii, Iraq, Owerri, Jos, and Zaria, Nigeria revealed that just two species of cockroaches (*Periplaneta americana *and* Blattella germanica*) were distributed in human habitations in their localities, out of the 3500 species of cockroaches discovered, 30 species have been adapted to human habitations [3].

*Periplaneta americana *was the most frequently trapped in Melong Subdivision. This remark is in agreement with the findings of Ejimadu et al. [24] in Jos, Nigeria but is in variance with claims done by Ebeling [26] that* B. germanica *reproduces faster than any other residential cockroach growing from egg to reproductive adult. The ability of* P. americana *to be trapped more easily than* B. germanica *and* B. orientalis *could be due to their tendencies of being more active in search of food and sites to lay their eggs [16] while* B. germanica *and* B. orientalis *look to be sedentary in their behavior.

According to the capture sites, most cockroaches were caught in the toilets, followed by those caught in kitchens and in the houses. Because of the constant moisture,* B. germanica *and* B. orientalis *were not present in toilets while* P. americana *were present in all sites. This can be explained by its great adaptation in diverse habitats.

The importance of cockroaches as carriers of parasitic worms, eggs, cysts, or oocysts was based on some reports about the presence of parasitic forms on or in cockroaches [27]. The infective rate of 47.39% recorded in cockroaches trapped from three locations of Melong Subdivision might be an indication of their filthy feeding habit which makes them efficient carriers of these pathogens as earlier reported by Greenberg [27], Etim et al. [17], and Nagham et al. [15]. This infective rate reported (47.39%) seemed to be higher than 18.41% observed by Ameh [25] in Zaria, Nigeria. However, it is lower than 67%, 77.52%, 98%, and 100% reported by Chan et al. [22], Bala and Sule [16], El-Sherbini and Gneidy [28], and Kassiri and Kazemi [21], respectively, in Owerri, Nigeria, Arkilla and Sokoto, Nigeria, Egypt, and Iran. These variances could be attributed to the state of the parasite considered in its life cycle. In the present study, only embryonated eggs and sporulated oocysts were considered because at that stage, they present a great potential to infest humans and other animals. But as mentioned by Okafor and Elenwo [29], these infective rates recorded in cockroaches are associated with the poor rural condition of the community and to people's habit of defecating in the bush due to the lack of adequate toilet facilities and other essential amenities.

*Ascaris *spp. and* Trichuris *spp., respectively, responsible for ascariasis and trichuriasis in human, were the most encountered irrespective of predilection site of fixation, sex, capture sites, and quarters. The highest transport rate of* Ascaris *spp. eggs observed in the present study can be explained by the fact that* Ascaris* eggs have an inner shell layer of lipoprotein nature which makes them more resistant to harsh environmental conditions and air-borne [30] compared to the eggs of other nematodes. Another reason is that* Ascaris *eggs can survive in adverse environmental conditions. It might also be due to the overdispersion of* Ascaris *eggs in the environment as a single female* Ascaris *lays relatively large number of eggs (200,000 eggs/day) [31]. Relatively lower rates of transport of* Trichuris *eggs observed in this study might be due to their minimal dispersion as a single female* Trichuris *liberates relatively less numbers of eggs and also due to the easy destruction of embryonated eggs by desiccation.

Associated with their breeding system in the study area, the presence of pig and others domesticated animal farms around households may favor in one hand the distribution of* Ascaris suum *and* Trichuris suis *eggs which are morphologically nondistinguishable from* Ascaris lumbricoides *and* Trichuris trichiura *eggs and, on the other hand, eggs of others animal parasites. One interesting finding in this study was the detection of* Capillaria* spp. and* Toxocara *spp. eggs. Parasitic eggs come from animals which were not identified in similar studies. It might be due to the animal husbandry in the city.

Hence, the observation of* Ascaris *spp. and* Trichuris *spp. ova in the body surface of cockroaches is in agreement with the proposition that cockroaches are seriously involved in the epidemiology of soil transmitted helminthes (STH) as earlier reported by Etim et al. [17].

From this study,* P. americana *was more qualified (*p* < 0.05) in the transport of parasites. This can be, as mentioned above, due to their adaptation in different environmental conditions and to their tendency of being more active in search of food and sites to lay their eggs [16]. Although the legs of many species of cockroaches are morphologically modified with comb like tubercles, spines or hairs, and useful during feeding and grooming processes, they are highly involved in the contamination of food and water in human dwellings [32, 33].

Based on capture sites, cockroaches caught from the toilets had more parasites because they were easily exposed to human faecal matter. As a result of their high mobility, they release these parasites (*Ascaris *spp.*, Trichuris *spp.*, Capillaria *spp., and Hookworm) carried on their bodies or within their alimentary canal on food or in drinking water when moving from toilet to kitchen and secondly from the kitchens to the houses; or on the contrary they enter in contact with other parasites like* Toxocara *spp. eggs and* Eimeria *spp. oocysts as observed in the present study.

Female cockroaches were significantly (*p* < 0.05) more vectorial than males. This may probably be due to their behavior which allows them to move more than the males in search of both food and sites to lay their eggs [16]. By this behavior, they come in contact with contaminated materials as they roam, making them more exposed to contact with pathogens.

Statistically, no significant difference was observed in the transport rate among the three-quarters. This simply allows the understanding that the parasites in these quarters have the same probability to be carried by cockroaches.

Mix infestations were observed among cockroach species and among different capture points. This result may probably explain the nonspecificity of cockroaches in their role of parasites vectors. Thus, the potential of cockroaches to transmit diseases should not be ignored or simply rejected but should be investigated further.

## 5. Conclusion

Cockroaches constitute an important reservoir for pathogens. Therefore, close contact with cockroaches especially in human dwellings should be discouraged. Due to the low standard of sanitation in Cameroon, especially in the Melong-Subdivision, there is a need to properly educate the population on the dangers associated with cockroaches and how to control them. Environmental hygiene is very necessary and should be encouraged in every locality, to reduce the population and bad effects of arthropod pests especially cockroaches in human surroundings.

## Figures and Tables

**Table 1:** Distribution of cockroach species by quarters and capture sites.

Sampling points	Areas/sites	Cockroach species *n* (%)			Total
		*Periplaneta americana*	*Blattella germanica*	*Blatta orientalis*	
Quarters	Nkongsoung	162 (19.20)	69 (8.18)	46 (5.45)	**277 (32.82)**
	Mélong centre	182 (21.56)	39 (4.62)	62 (7.35)	**283 (33.53)**
	Lèlem	149 (17.65)	73 (8.65)	62 (7.35)	**284 (33.65)**

Total		**282 (33.41)**	**277 (32.82)**	**285 (33.77)**	**844 (100)**

Captures sites	Toilets	282 (33.80)	0 (0.00)	0 (0.00)	**282 (33.41)**
	Kitchens	131 (15.52)	62 (7.35)	84 (9.95)	**277 (32.82)**
	Houses	80 (9.48)	119 (14.10)	86 (10.19)	**285 (33.77)**

Total		**493 (58.41)**	**181 (21.45)**	**170 (20.14)**	**844 (100)**

**Table 2:** Prevalence of parasites grouped by sites of infestation and by sexes of cockroaches.

Parasite	Sites of infestation		Total	Sexes		Total
	External surface	Internal surface		Male	Female	
*Ascaris* spp.	240 (28.44)^a^	179 (21.21)^b^	**419 (49.64)**	101 (11.97)^a^	184 (21.80)^b^	**285 (33.76)**
*Trichuris* spp.	81 (9.60)^a^	58 (6.87)^b^	**139 (16.46)**	47 (5.57)^a^	54 (6.40)^a^	**101 (11.97)**
*Capillaria* spp.	46 (5.45)^a^	31 (3.67)^a^	**77 (9.12)**	28 (3.32)^a^	24 (2.84)^a^	**52 (6.16)**
*Toxocara* spp.	38 (4.50)^a^	28 (3.32)^a^	**66 (7.82)**	26 (3.08)^a^	15 (1.78)^a^	**41 (4.86)**
Hookworm	30 (3.55)^a^	12 (1.42)^b^	**42 (4.98)**	13 (1.54)^a^	28 (3.32)^b^	**41 (4.86)**
*Eimeria* spp.	23 (2.73)^a^	17 (2.01)^a^	**40 (4.74)**	12 (1.42)^a^	11 (1.30)^a^	**23 (2.73)**

a and b on the same column = significant at 0.05 - a, a on the same line = nonsignificant at 0.05.

**Table 3:** Parasite recovery rate from cockroaches in different quarter and capture sites.

Sampling points	Areas/sites	Prevalence (%)					
		*Ascaris* spp.	*Trichuris* spp.	*Capillaria* spp.	*Toxocara* spp.	Hookworms	*Eimeria* spp.
Quarter	Nkongsoung	118 (13.98)^b^	36 (4.27)^a^	6 (0.71)^b^	8 (0.95)^b^	7(0.83)^b^	2 (0.2)^b^
	Mélong centre	77 (9.12)^a^	22 (2.61)^a^	14 (1.66)^a,b^	15 (1.78)^a,b^	11 (1.31)^a,b^	7 (0.83)^a.b^
	Lèlem	90 (10.66)^a^	43 (5.09)^a^	21 (2.45)^a^	28 (3.2)^a^	24 (2.83)^a^	14 (1.30)^a^

Total		**285 (33.77)**	**101 (11.97)**	**52 (6.16)**	**(4.86)**	**41 (4.86)**	**23 (2.73)**

Capture sites	Toilets	145 (17.19)^a^	63 (7.46)^b^	20 (2.37)^a^	0 (0.00)^b^	32 (3.79)^b^	0 (0.00)^b^
	Kitchens	108 (12.79)^a^	24 (2.84)^a^	21 (2.49)^a^	17 (2.01)^a^	7 (0.81)^a^	14 (1.66)^a^
	Houses	35 (4.15)^b^	14 (1.66)^a^	11 (1.30)^a^	23 (2.73)^a^	2 (0.24)^a^	9 (1.07)^a^

Total		**285 (33.77)**	**101 (11.97)**	**52 (6.16)**	**41 (4.86)**	**41 (4.86)**	**23 (2.73)**

a and b on the same column = significant at 0.05 - a, a on the same column = non significant at 0.05.
